# Cell type specific cannabinoid CB1 receptor distribution across the human and non-human primate cortex

**DOI:** 10.1038/s41598-022-13724-x

**Published:** 2022-06-10

**Authors:** Shinnyi Chou, Tejis Ranganath, Kenneth N. Fish, David A. Lewis, Robert A. Sweet

**Affiliations:** Department of Psychiatry, University of Pittsburgh Medical Center, Pittsburgh, USA

**Keywords:** Translational research, Neurotransmitters

## Abstract

Alterations in cannabinoid CB1 receptor (CB1R) are implicated in various psychiatric disorders. CB1R participates in both depolarization induced suppression of inhibition (DSI) and depolarization induced suppression of excitation (DSE), suggesting its involvement in regulating excitatory and inhibitory (E/I) balance. Prior studies examining neuronal cell type specific CB1R distribution have been conducted near exclusively within rodents. Identification of these distribution patterns within the human and non-human primate cortex is essential to increase our insight into its function. Using co-labeling immunohistochemistry and fluorescent microscopy, we examined CB1R protein levels within excitatory and inhibitory boutons of male human and non-human primate prefrontal cortex and auditory cortices, regions involved in the behavioral effects of exogenous cannabinoid exposures. We found that CB1R was present in both bouton populations within all brain regions examined in both species. Significantly higher CB1R levels were found within inhibitory than within excitatory boutons across all regions in both species, although the cell type by brain region interactions differed between the two species. Our results support the importance of conducting more in-depth CB1R examinations to understand how cell type and brain region dependent differences contribute to regional E/I balance regulation, and how aberrations in CB1R distribution may contribute to pathology.

## Introduction

Cannabis, a drug derived from the Cannabis plant, is the most widely used illicit psychoactive substance worldwide, with a steady increase in usage over the past decade in the setting of increased legalization of recreational marijuana use^1,2^. Cannabis use has been associated with various psychiatric comorbidities^3^, including substance use disorders and schizophrenia^4^.

The cannabinoid CB1 receptor (CB1R) is the main receptor target in the brain for tetrahydrocannabinol (THC), the major psychoactive substance in cannabis^5^. CB1R is one of the most prevalent G-protein coupled receptors (GPCR) in the central nervous system^6^. Functionally, its main mechanism appears to be depolarization dependent presynaptic modulation of inhibitory neurotransmitter release through G_i/o_ coupling^7,8^, a phenomenon termed depolarization-induced suppression of inhibition (DSI).

While previously considered nearly exclusively localized to presynaptic terminals of cholecystokinin-expressing (CCK) GABAergic interneurons^9–12^, CB1R has more recently been identified in other cell types in rodents^13–15^. In addition, CB1R has been found in excitatory populations such as glutamatergic neurons within the cerebellum^16^, striatum^17^, ventral tegmental area^18^, amygdala^19^, hippocampus^16,19^, and neocortex^20^. CB1R’s activity at presynaptic excitatory terminals results in depolarization induced suppression of excitation (DSE)^14^, a phenomenon analogous to DSI. Thus, CB1R’s influence across excitatory and inhibitory (E/I) neuron synapses posits its importance in regulating cortical E/I balance^21^.

It is important to note that neuronal cell type specific CB1R distribution studies thus far have been conducted near exclusively within rodents^22^. Considering the aforementioned associations between cannabis exposure, which modulates CB1R activity, and various psychiatric disorders, identifying normative cell type specific distribution of CB1R within humans is essential to increasing our understanding of these disorders.

Given the paucity of such studies, the current work investigated normative distribution of cell type specific CB1R within two representative cortical regions using healthy postmortem human and non-human primate samples. Inclusion of non-human primate samples represents an important comparison, given its value in future translational studies. The prefrontal and auditory cortices were chosen given their involvement in the behavioral effects of exogenous cannabinoid exposures in humans, as well as notable differences in CB1R levels between the two regions in prior reports^23^. We hypothesize the presence of CB1R within both excitatory and inhibitory neuronal populations in all brain regions, with differential distribution of CB1R between these cell types.

## Methods

### Monkey tissue

All monkey brain specimen sources (long-tailed macaque; Macaca fascicularis) were acquired from an existing bank of para-formaldehyde fixed monkey brain tissues. Monkeys were previously housed in the University of Pittsburgh Plum Animal Facility, and husbandry and animal care were performed daily by University of Pittsburgh Division of Laboratory Animal Research staff. Animals were euthanized in 1990 and brain sections carefully processed and stored until use as described below.

Brain specimens from two adult male monkeys (5–6 years of age) were utilized for fluorescent microscopy. Both animals were experimentally naïve prior to be being euthanized. At the ages chosen, the monkeys are sexually mature, past the period of development pruning of cortical synapses, and considered to be young adults.

Monkeys were deeply anesthetized with ketamine hydrochloride (25 mg/kg) and sodium pentobarbital (30 mg/kg), intubated, mechanically ventilated with 28% O2/air, and perfused transcardially with ice-cold 1% paraformaldehyde in 0.1 M phosphate buffer (pH 7.4) followed by 4% paraformaldehyde in phosphate buffer, as previously described^23^. Brains were immediately removed, blocked into 5–6-mm-thick coronal blocks, and postfixed for 6 h in phosphate-buffered 4% paraformaldehyde at 4 C. Tissue blocks were subsequently immersed in cold sucrose solutions of increasing concentrations (12%, 16%, and 18%) then stored at − 30 °C in a cryoprotectant solution containing 30% glycerol and 30% ethylene glycol in diluted phosphate buffer until sectioning. Tissue blocks from the left hemisphere containing the superior frontal gyrus (dorsolateral prefrontal cortex) and superior temporal gyrus (primary and association auditory cortex) were sectioned coronally at 40 um to exhaustion on a cryostat, and every 10th section was stained for Nissl substance with thionin to serve as anatomical references. Unstained sections were stored until processed for immunohistochemistry at − 30 °C in the same cryoprotectant solution as above.

All methods, including housing and experimental protocols, were carried out in accordance with relevant guidelines and regulations from the United States Department of Agriculture and National Institutes of Health guidelines. All experimental protocols were approved by the University of Pittsburgh’s Institutional Animal Care and Use Committee (IACUC). All methods are reported in accordance with ARRIVE guidelines.

### Human tissue

All human brain specimen sources were collected during autopsies conducted at the Allegheny County Medical Examiner’s Office, Pittsburgh, PA. Samples from three adult male human subjects (48–55 years of age; postmortem interval [PMI] 6–12 h) were obtained following informed consent for brain donation from the next of kin.

None of the subjects had a history of psychiatric or neurologic disorders as determined by information obtained from clinical records and a structured interview conducted with a surviving relative by an independent committee of experienced research clinicians. This included any known history of cannabis use or use disorders from interview and review. See Table 1 for subject demographics. Following retrieval of brain specimens, the left hemisphere was cut into 1.0–2.0 cm-thick coronal blocks and fixed for 48 h in phosphate-buffered 4% paraformaldehyde at 4 C. Tissue blocks were subsequently immersed in graded cold sucrose solutions then stored at − 30 °C in the cryoprotectant solution as described previously until sectioning.

**Table 1:** Demographic information of subjects from which postmortem human brain samples were obtained.

Subject ID	Sex	Race	Age	PMI
Hu681	Male	White	51	11.6
Hu857	Male	White	48	16.6
Hu1284	Male	White	55	6.4

PMI = postmortem interval between which subjects were deceased and samples were harvested measured in hours.

Tissue blocks containing the superior frontal gyrus (dorsolateral prefrontal cortex) and superior temporal gyrus (containing the primary auditory cortex) were sectioned coronally at 40 um on a cryostat, and every 40th section was stained for Nissl substance with thionin to serve as anatomical references. Unstained sections were stored until processed for immunohistochemistry at − 30 °C in the same cryoprotectant solution as above.

All methods were carried out in accordance with relevant guidelines and regulations from the University of Pittsburgh’s Committee for the Oversight of Research and Clinical Trials Involving Decedents. All experimental protocols were approved by the University of Pittsburgh’s Institutional Review Board for Biomedical Research.

### Immunohistochemistry

For each monkey and human subject, a free-floating tissue section containing the region of interest, identified using neighboring Nissl-stained sections was used. Sections were washed in 0.1 M phosphate-buffered saline (PBS) then incubated for 75 min in 0.01 M sodium citrate solution at 80 °C to retrieve antigens and enhance immunohistochemical labeling^24^. After cooling to room temperature (RT), sections were immersed in 1% sodium borohydride for 30 min at RT to reduce background autofluorescence^25^. This was followed by membrane permeabilization with 0.3% Triton X-100 in PBS for 30 min at RT. Sections were then blocked with 20% normal goat serum in PBS for 2 h at RT to reduce nonspecific antibody binding, followed by incubation for 72 h at 4 °C in PBS containing 2% normal goat serum and primary antibodies.

The primary antibodies used include monoclonal mouse anti-vGAT antibody (1:500; Synaptic Systems, Göttingen, Germany; product # 131,011)—which label intracortical inhibitory boutons; polyclonal guinea pig anti-vGlut1 antibody (1:500; Millipore Sigma, Burlington, MA; product # AB5905)—which label intracortical excitatory boutons, and polyclonal rabbit anti-CB1R antibody (1:2000; Synaptic Systems, Göttingen, Germany; product # 258,003). We previously demonstrated successful and specific vGAT and vGlut1 labeling in human and non-human primate postmortem studies using the aforementioned antibodies^26–29^. The CB1R antibody demonstrated successful co-labeling with both vGAT and vGlut1 in neuronal cultures^30^. In addition, vGAT antibody specificity was validated through knockout tissue samples^31^, vGlut1 antibody through pre-adsorption control (Millipore certificate of analysis, 2016), and CB1R antibody through knockout samples^32^.

Post primary antibody incubation, sections were rinsed for 2 h (4 × 30 min) in PBS and incubated for 24 h in PBS containing 2% normal goat serum and secondary antibodies (goat host) conjugated to Alexa 488 (1:500; vGlut1), Alexa 568 (1:500; CB1R) and Alexa 647 (1:500; vGAT; Invitrogen, Grand Island, NY, for all Alexa antibodies) at 4 °C. Sections were then rinsed for 2 h in PBS (four rinses of 30 min each), mounted on gelatin subbed slides, cover slipped (ProLong Gold antifade reagent, Invitrogen), sealed with clear nail polish along coverslip edges, and stored at 4 °C until imaged.

### Tissue sampling

Sampling procedure was as previously described^33,34^. Contours outlining each cortical section were drawn in Stereo Investigator version 8 (MicroBrightField Inc., Natick, MA). To ensure representative sampling of the complete gray matter in each brain region, for each section, image stacks were obtained from six to ten randomly chosen sites for each cortical layer. Layers were determined by measurements made in nearby Nissl-stained sections. At each sampling site, tissue thickness (z-axis depth) was measured and divided by 40 µm (original sectioned thickness) to correct for shrinkage during tissue processing.

### Confocal microscopy

Microscopy equipment and capturing parameters were as previously described^35^. Data were collected using a 60 × 1.40 numerical aperture supercorrected oil immersion objective mounted on an Olympus BX51Wl upright microscope (Olympus America Inc., Center Valley, PA) equipped with an Olympus spinning disk confocal unit, Hamamatsu Orca R2 camera (Hamamatsu, Bridgewater, NJ), MBF CX9000 front mounted digital camera (MicroBrightField Inc., Natick, MA), BioPrecision2 XYZ motorized stage with linear XYZ encoders (Ludl Electronic Products Ltd., Hawthorne, NY), excitation and emission filter wheels (Ludl Electronic Products Ltd., Hawthorne, NY), Sedat Quad 89,000 filter set (Chroma Technology Corp., Bellows Falls, VT), and a Lumen 220 metal halide lamp (Prior Scientific, Rockland, MA).

The equipment was controlled by SlideBook 6.0 (Intelligent Imaging Innovations, Inc., Denver, CO), which was the same software used for post-image processing. Three-dimensional image stacks (two-dimensional images successively captured at intervals separated by 0.25 mm in the z-dimension) were acquired with a total depth spanning 20% of tissue thickness, starting from the plane furthest away from the coverglass and stepping up until tissue surface was reached. Image planes were 512 × 512 pixels (55 × 55 µm) in the XY dimension. Stacks were collected using optimal exposure settings (i.e., those that yielded the greatest dynamic range possible for the camera with no saturated pixels). Z-axis positions were normalized to original section thickness, and exposures were normalized for each image capture, during post-image processing prior to analysis.

### Image processing

Images were processed as previously described^35,36^ using SlideBook with keystrokes automated by Automation Anywhere software (Automation Anywhere, Inc., San Jose, CA). Image stacks were deconvolved using AutoQuant’s blind deconvolution algorithm (MediaCybernetics, Rockville, MD). After deconvolution, separate Gaussian channels were made for each deconvolved channel by calculating a difference of Gaussians filters generated using sigma values of 0.7 and 2. These Gaussian channels, which enhance the demarcation of immunofluorescence edges, were used for data segmentation.

Segmentation of the Gaussian channels was performed using an iterative combined intensity/morphologic thresholding algorithm as previously described^26^. After obtaining an initial value for iterative segmentation for each channel derived using Otsu’s method within Slidebook, each subsequent iteration increased threshold by 50 Gy levels, and object masks were size gated within a range of 0.03 and 2.0 µm^3^. After each segmentation, masked objects were merged with prior iterations, with the final resulting masks copied back onto the original deconvolved channels (i.e., without Gaussian subtraction), to obtain pixel intensity information. Lipofuscin, an autofluorescent lysosomal degradation product, which may confound quantitative fluorescence measures in human postmortem tissues, was excluded by imaging of lipofuscin using a separate channel at a constant exposure time across all sections.

After generating vGAT and vGlut1 masks encompassing individual boutons, mean CB1R intensity underneath each masked object was obtained for each bouton type. These mean intensities were then averaged across all boutons per sampled site for each bouton type. The resulting cell type specific mean CB1R intensity value per sampled site, measured in analog-to-digital units (ADU), served as the dependent measure.

### Statistical analysis

Prior to conducting analyses, fluorescent intensity data were filtered to ensure accurate representation of receptor labeling. Specifically, based upon examination of antibody signal penetrance across tissue thickness, only those objects falling within 2–4 µm from tissue surface after correcting for tissue shrinkage were included in the analyses. In addition, to prevent potential spherical aberration confounding intensity measurements, signals falling within the upper and lower 2% of the x- and y-dimensions were excluded from analyses. To further ensure accurate capture of cell type specific measurements, objects overlapping both vGlut1 and vGAT masks were excluded from analysis.

All data were expressed as mean ± standard error of the mean (SEM). To analyze brain region and cell type specific differences in CB1R intensity, a mixed model analysis of variance was used to account for nested correlations (multiple cell types within each sampled site and multiple sampled sites within each brain region). Brain region, cell type, and brain region by cell type are entered as fixed effects, and subject is input as a random effect. Significant differences were followed by post hoc Bonferroni tests to correct for the increased risk of a type I error when making multiple statistical tests. For all analyses, *p* < 0.05 was considered statistically significant.

## Results

### Cell type and brain region specific distributions of CB1R relative protein level in the monkey and human cortex

**Figure 1:**
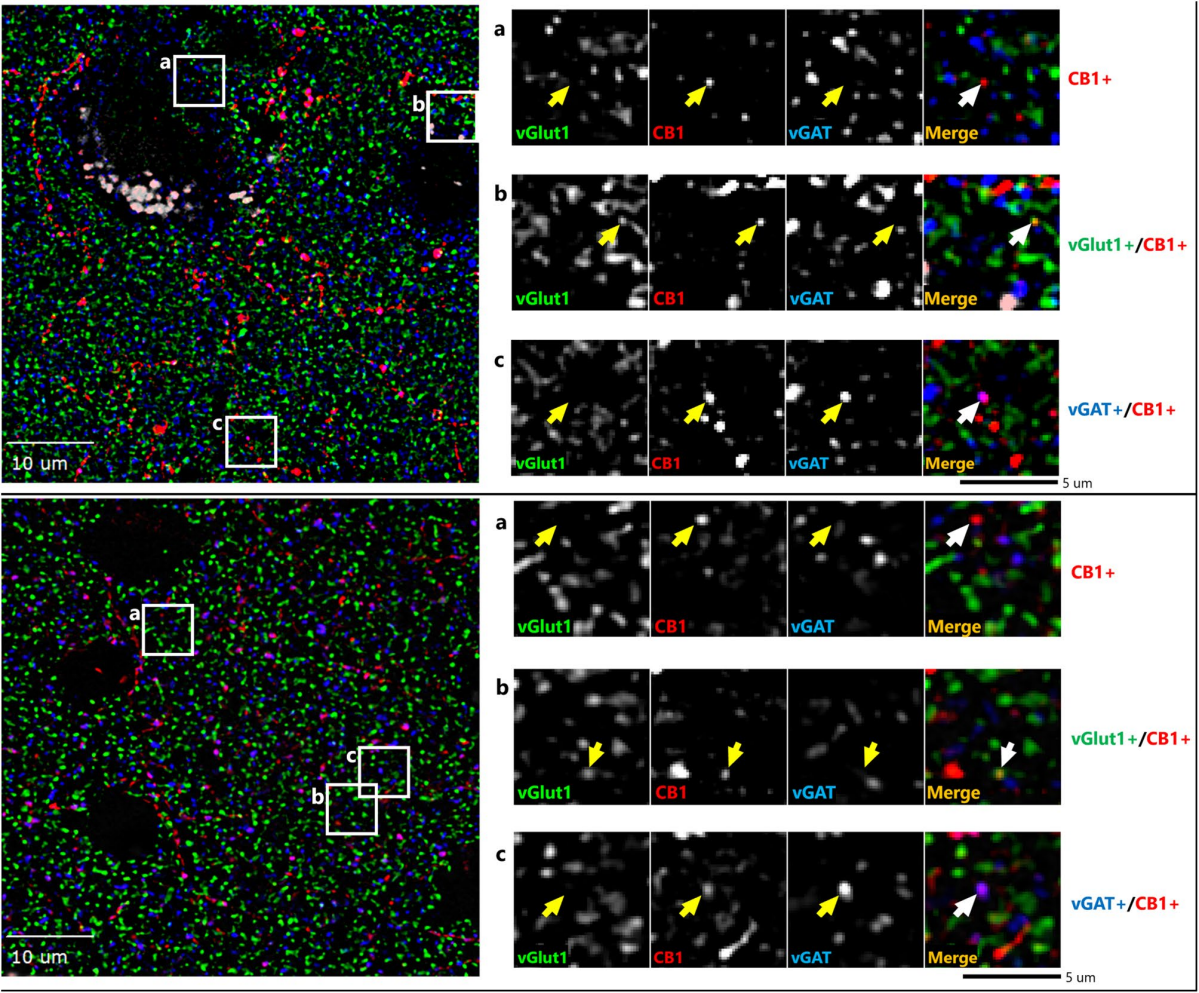
Sample micrograph of immunohistochemical staining in both human (top) and monkey (bottom) cortex. Left panels: Puncta with vGlut1 + (green), vGAT + (blue) & CB1R + (red) immunoreactivity are distributed throughout the image field. Right panels: Enlarged images with arrows identifying puncta with antibody immunoreactivity toward a single or multiple proteins.

Qualitative review of our fluorescent micrographs revealed CB1R labeling in both inhibitory (vGAT +) and excitatory (vGlut1 +) boutons in both monkey and human cortex (Fig. 1). As expected, CB1R immunoreactivity was also localized to soma and apparent axons and dendrites, and these CB1R-immunoreactive (IR +) fluorescent signals did not co-label with vGAT-IR + or vGlut1-IR + boutons.

**Figure 2:**
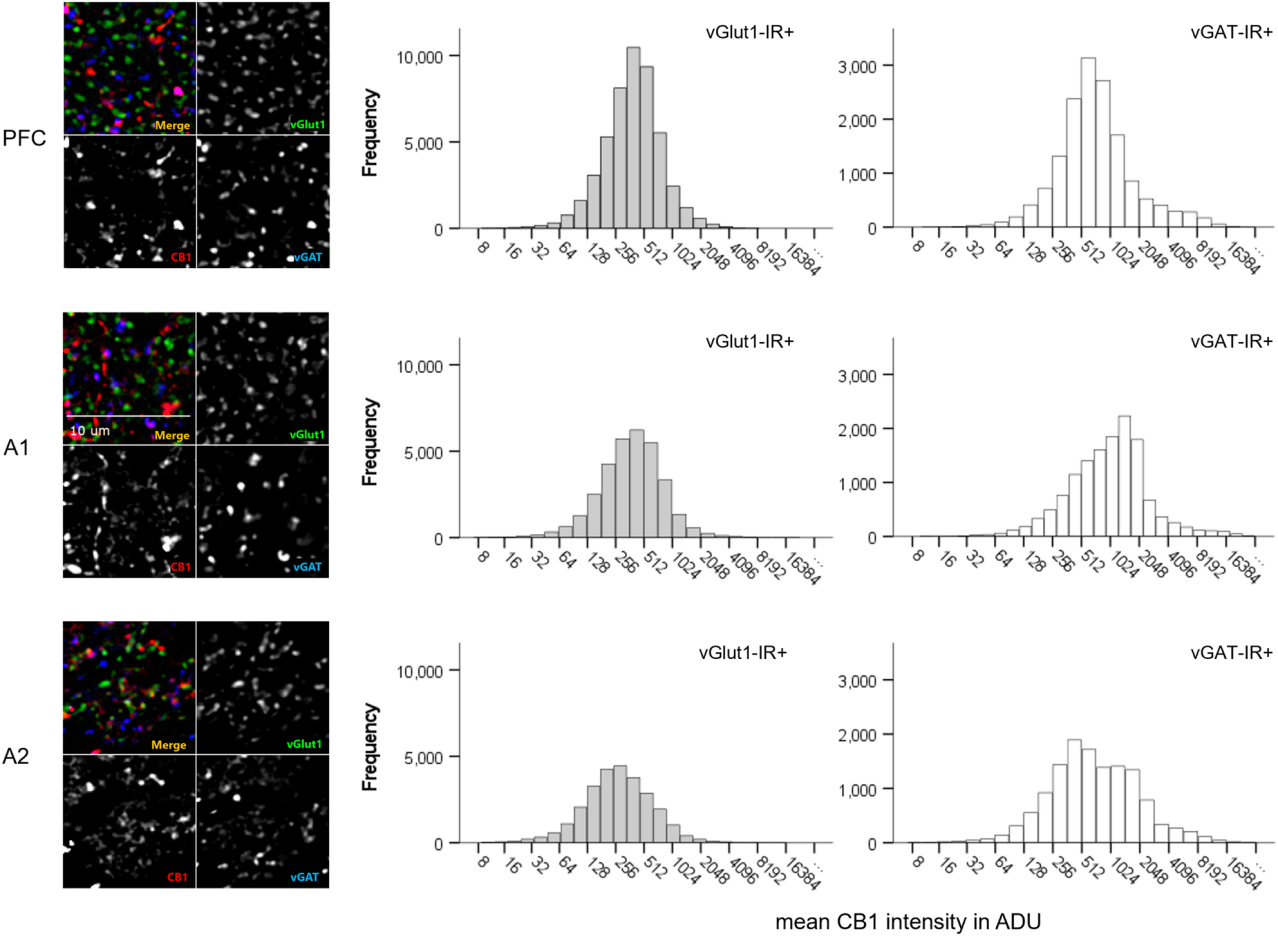
Mean CB1R intensity frequency histograms for excitatory (vGlut1-IR +) and inhibitory (vGAT-IR +) cell types across three brain regions of the monkey cortex, measured in analogue-to-digital units (ADU). PFC = prefrontal cortex, A1 = primary auditory cortex, A2 = association auditory cortex. Scale bar can be seen in the sample image for A1.

**Figure 3:**
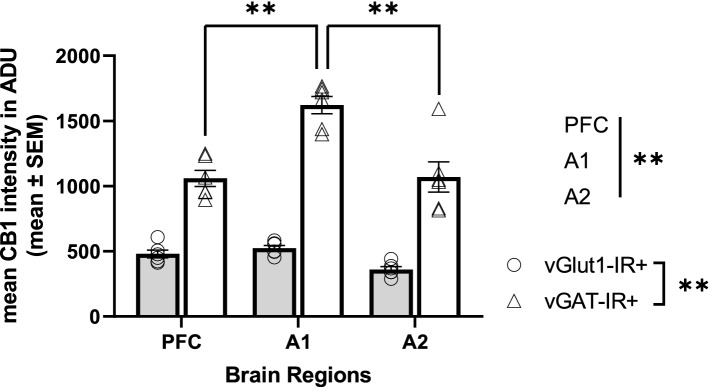
Mean CB1R intensity within excitatory (vGlut1-IR +) and inhibitory (vGAT-IR +) cell types across three brain regions of the monkey cortex. Each individual data point represents mean intensity averaged across all sampled sites across a cortical layer. There was a main effect of cell type, *p* < 0.001, main effect of brain region, *p* < 0.001, and a significant cell type × brain region interaction, *p* < 0.001. While there were no differences in mean CB1R intensity within excitatory populations across brain regions, post hoc analysis indicated mean CB1R intensity within inhibitory populations was highest in the primary auditory cortex, followed by the association auditory cortex, with lowest mean CB1R intensity within inhibitory populations in the PFC, *p* < 0.001. Straight lines indicate main effects across groups. Brackets indicate post hoc pairwise comparisons. **p < 0.001. Error bar denotes SEM. PFC = prefrontal cortex, A1 = primary auditory cortex, A2 = association auditory cortex.

CB1R intensity histograms reflect notable differences across brain regions and cell types within monkey cortical samples (Fig. 2). Statistical analysis revealed a main effect of cell type, F(1,591) = 882.015, *p* < 0.001 and brain regions, F(2,591) = 23.658, *p* < 0.001 (Fig. 3). Across all three cortical regions, mean CB1R fluorescent intensity was significantly higher within vGAT-IR + boutons compared to vGlut1-IR + boutons. See Table 2 for CB1R mean intensity values in ADU.

**Table 2:** CB1R mean intensity in analogue-to-digital units (ADU) across cell type and brain region for monkey and human samples.

Species	Cell type	Brain region		
		PFC	A1	A2
Monkey	Excitatory (vGlut1-IR +)	480.984 ± 219.050	449.677 ± 219.852	452.872 ± 219.944
	Inhibitory (vGAT-IR +)	1077.951 ± 219.050	1547.226 ± 219.852	1187.688 ± 219.944
Human	Excitatory (vGlut1-IR +)	391.417 ± 109.560	621.843 ± 109.471	556.900 ± 109.479
	Inhibitory (vGAT-IR +)	598.244 ± 109.560	948.501 ± 109.471	940.308 ± 109.479

Values are represented as mean ± SEM. PFC = prefrontal cortex, A1 = primary auditory cortex, A2 = association auditory cortex.

**Figure 4:**
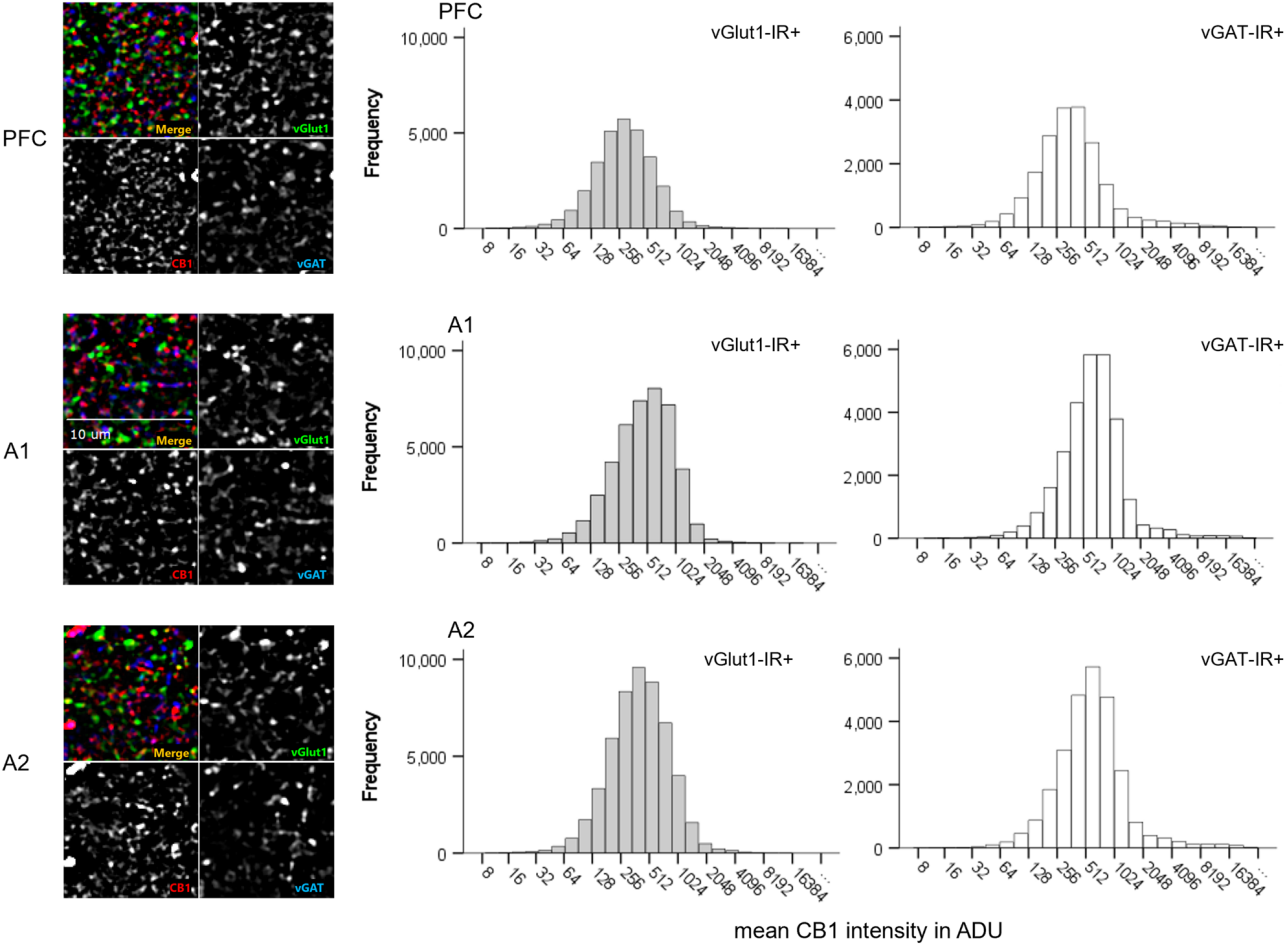
Mean CB1R intensity frequency histograms for excitatory (vGlut1-IR +) and inhibitory (vGAT-IR +) cell types across three brain regions of the human cortex, measured in analogue-to-digital units (ADU). PFC = prefrontal cortex, A1 = primary auditory cortex, A2 = association auditory cortex. Scale bar can be seen in the sample image for A1.

**Figure 5:**
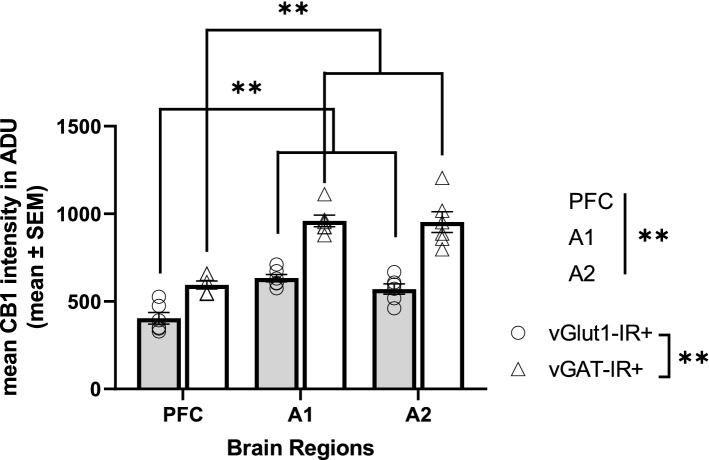
Mean CB1R intensity within excitatory (vGlut1-IR +) and inhibitory (vGAT1-IR +) cell types across three brain regions of the postmortem human cortex. Each individual data point represents mean intensity averaged across all sampled sites across a cortical layer. There was a main effect of cell type, *p* < 0.001, main effect of brain region, *p* < 0.001, and a significant cell type × brain region interaction, *p* < 0.001. Post hoc analysis indicated significantly lower mean CB1R intensity within the PFC for both excitatory and inhibitory populations compared to the primary and association auditory cortex, *p* < 0.001 for all comparisons. Straight lines indicate main effects across groups. Brackets indicate post hoc pairwise comparisons. **p < 0.001. Error bar denotes SEM. PFC = prefrontal cortex, A1 = primary auditory cortex, A2 = association auditory cortex.

Similarly, CB1R intensity frequency histograms reflect notable differences across brain regions and cell types within postmortem human cortical samples (Fig. 4). We identified a main effect of cell type, F(1,1055) = 408.243, *p* < 0.001 and brain regions, F(2,1055) = 143.101, *p* < 0.001 (Fig. 5). Across all three cortical regions, mean CB1R fluorescent intensity appears significantly higher within vGAT-IR + boutons compared to vGlut-IR + boutons. See Table 2 for CB1R mean intensity values in ADU.

### Cell type by brain region interactions in CB1R relative protein level distributions within the monkey and human cortex

Within monkey sections, results indicated a significant cell type by brain region interaction, F(2,591) = 30.582, *p* < 0.001 (Fig. 3). Post-hoc tests revealed that the significant region dependent differences in mean CB1R intensity was only evident within vGAT-IR + boutons, F(2,591) = 53.924, *p* < 0.001, while within vGlut1-IR + boutons no significant mean CB1R intensity differences were identified across brain regions, F(2,591) = 0.297, *p* = 0.743. Additional pair-wise comparison of vGAT-IR + boutons indicated significantly higher mean CB1R intensity within vGAT-IR + boutons in the primary auditory cortex compared to both the PFC and association auditory cortex (both *p* < 0.001).

Similarly, within postmortem human cortical samples, there was a significant cell type by brain region interaction, F(2,1055) = 11.650, *p* < 0.001 (Fig. 5). Post-hoc tests revealed that the significant region dependent differences in mean CB1R intensity was evident within both vGlut1-IR + , F(2,1055) = 40.581, *p* < 0.001, and vGAT-IR + boutons, F(2,1055) = 114.017, *p* < 0.001. Additional pair-wise comparisons indicated significantly lower CB1R mean intensity within the PFC compared to both the primary and association auditory cortex when examining both vGlut1-IR + and vGAT-IR + boutons (*p* < 0.001 for all comparisons).

## Discussion

### Summary of current findings

The present work assessed cell type and region-specific distribution of the CB1R receptor across the human and non-human primate cortex. We focused on two representative cortical regions, the prefrontal and auditory cortices, given their involvement in the behavioral effects of exogenous cannabinoid exposures in humans. Importantly, these two regions provide valuable contrast, as prior reports noted higher detectable CB1R immunoreactivity density within the prefrontal cortex relative to the primary auditory cortex^23^. In both monkey and human samples, we found that CB1R was present in both excitatory and inhibitory neuron populations within the PFC, primary auditory cortex, and association auditory cortex, with significantly higher levels within inhibitory populations. Interestingly, we identified differences in cell type specific regional distributions between the two species. Within the excitatory boutons, mean CB1R intensity was consistent across brain regions in monkey samples, while it was lowest in the PFC compared to the auditory regions in human samples. In contrast, within the inhibitory boutons, mean CB1R intensity was lower in the PFC compared to other regions, and highest in the primary auditory cortex for both species.

### Cortical cell type specific distributions of CB1R

Although CB1R has been identified in various subcellular localizations, it is most well-known to inhibit neurotransmitter release through activation of presynaptcally located receptors^37,38^. Depending on the cell type this presynaptic localization may result in either DSI or DSE. In the current study, mean CB1R intensity was higher in inhibitory than excitatory boutons. This is in concordance with previous rodent studies examining various cortical and noncortical regions such as the sensorimotor cortex, hippocampus, and cerebellum^16,39^.

It is likely that protein level differences between inhibitory and excitatory boutons directly contribute to functional distinctions. For example, in paired patch-clamp recordings of CA1 pyramidal neurons and CCK + basket cells, tonic activation of CB1R by endocannabinoids was present in rat hippocampal slices and associated with persistent suppression of GABA transmission, which was rescued by the application of the CB1R antagonist AM251^40^. However, this intrinsic phenomenon does not appear to be equally present at excitatory synapses. Using mice hypothalamic slices containing proopiomelanocortin (POMC) neurons, Hentges et al., 2005 observed a similar reduction in GABA transmission under basal endocannabinoid release, which was also attenuated with exposure to AM251^41^. Neither basal endocannabinoid activity nor CB1R antagonism affected excitatory transmission in their study. However, exogenous CB1R agonism with WIN 55,212–2 led to reductions in both inhibitory and excitatory synaptic currents, consistent with a lower CB1R availability (and thus a higher threshold for DSE) at excitatory compared to inhibitory synapses. The effects of endocannabinoid induced DSI at GABAergic synapses, its enhancement with exposure to WIN 55,212–2, and its abolishment with administration of the CB1R antagonist rimonabant, have also been demonstrated in human neocortical slices through patch-clamp recordings^42^. However, studies to date have not examined CB1R induced DSE using human cortical samples.

### Regional and cell type by region interactions of CB1R

In the current study, we identified the presence of CB1R within both excitatory and inhibitory neuronal populations in the human and non-human primate PFC and auditory cortices. The role of CB1R activities in the PFC and its regulation of cognitive functions have been explored by various groups. It is established that CB1R plays a critical role in regulating E/I balance within the PFC^21^, and agonist exposures may reduce total protein levels^43^, as well as modulate circuitry functions and behaviors^44,45^.

Our results identifying higher mean CB1R intensity within the primary auditory cortex compared to the prefrontal cortex in human samples suggest that cannabis exposure may also exert strong modulations on auditory functions. Indeed, anecdotal human accounts of altered auditory experiences under the influence of marijuana are well known^46^. More recently, controlled experiments have begun to elucidate mechanisms by which exogenous cannabinoid exposure influences auditory cortical activity.

For example, when administered capsules containing standardized cannabis extracts, subjects exposed to a pure tone deviance task demonstrated a significant negative correlation between mismatch negativity amplitudes and the plasma concentration of the THC metabolite 11-OH-THC^47^. In addition, using a standardized cannabis inhalation protocol, subjects demonstrated reduced auditory cortex activity in response to music stimuli compared to controls^48^. Similar findings of reduced auditory cortex activation in response to neutral words read as auditory stimuli were seen in subjects administered THC capsules as well^49^.

The behavioral effects of CB1R agonism within a region are likely related to the balance of cell type specific CB1R activations induced. Indeed, a recent study notes that glutamatergic CB1R activation in the forebrain led to THC-induced hypothermia in mice, while GABAergic CB1R activation led to THC-induced hyperlocomotion^50^. Similarly, in global CB1R deletion animals, rescue of dorsal telencephalic glutamatergic CB1R restored hippocampal DSE and protected animals against chemically induced seizures^51^, a phenomenon not seen with rescue of GABAergic CB1R^52^.

In addition, through measuring isolated excitatory and inhibitory miniature postsynaptic currents, previous studies have observed that the net effect of CB1R agonism in mouse PFC is to shift the E/I balance towards excitation^21^. Given the regional differences observed in human cell type specific CB1R distribution, we expect that CB1R activation may exert different magnitudes of depolarization induced suppressions of neurotransmission across regions, leading to region dependent E/I regulation. Importantly, given the different cell type by brain region interactions observed across species, we suggest that exogenous cannabinoid exposures may lead to differing region-specific shifts in E/I balance and behavioral consequences across these two species—an important consideration when choosing a translational model.

### Limitations

While the present study increases our understanding of CB1R’s cell type specific distribution, the limitation in subject selection means the results may not be generalizable to the female sex. In addition, our subject selection was limited to adult samples, and it is unclear whether cell type specific CB1R distribution varies across developmental stages^53^, which may have implications for differential effects of exogenous cannabis exposure on alterations of E/I balance. Although we chose three cortical regions that demonstrated differing levels of CB1R expression in prior non-human primate studies, the lack of global assessment precludes us from generalizing current results throughout the entire cortex, particularly for areas previously shown to have high (e.g., dentate gyrus) or low (e.g., primary visual cortex) CB1R expression. Furthermore, this study provides a broad overview of CB1R distributions in excitatory and inhibitory neuron populations, with the understanding that each of these cell populations encompass highly complex subpopulations of neurons with specific functions. Given the choice of assessing vGlut1 and vGAT boutons, we were unable to confirm the cell types of CB1R-IR + puncta that did not demonstrate co-labeling, and it is likely these non-vGlut1 and non-vGAT puncta represent a variety of cell types such as vGlut2-expressing glutamatergic neurons^54^. Of note, recent studies have also demonstrated the presence of CB1R in non-GABAergic and non-glutamatergic cell types (e.g., astrocytes, dopaminergic neurons)^55^, as well as in a variety of different subcellular organelles (e.g., lysosomes, endosomes, mitochondria)^56^, and future studies should expand into the investigation of subcellular CB1R localization in postmortem human brain samples.

### Conclusion and future directions

The current study confirms the presence of CB1R within the terminals of both excitatory and inhibitory neuronal populations in non-human primate and human postmortem brain samples. We identified lower overall CB1R intensity in excitatory compared to inhibitory bouton populations that was conserved across species. In addition, we identified differences in cell type specific CB1R distribution across brain regions between non-human primate and human postmortem brain samples—namely a regional difference in excitatory bouton CB1R intensity in human samples that was not present in monkey samples. These differences suggest variations in the regulation of E/I balance by CB1R across brain regions and that this regional variation differs between humans and non-human primates. Though limitations exist, as discussed above, these results support the importance of conducting more in-depth CB1R examinations to understand how these parameters interact to result in the final E/I output. Such studies may include quantitative comparisons of cell type and region dependent constitutive and ligand-induced DSI and DSE changes, and subsequent modulations of E/I balance. Additional consideration should be given to translating our methodology to investigation of cell type specific patterns of CB1R expression in additional neuronal subtypes, during neurodevelopment, and in individuals in whom cannabis use may exacerbate an underlying pathology, such as in individuals with schizophrenia^57^.

## Data Availability

Data supporting the results reported in the article is directly available upon request.
